# IL-10 Deficiency Accelerates Type 1 Diabetes Development *via* Modulation of Innate and Adaptive Immune Cells and Gut Microbiota in *BDC2.5* NOD Mice

**DOI:** 10.3389/fimmu.2021.702955

**Published:** 2021-07-30

**Authors:** Juan Huang, Qiyuan Tan, Ningwen Tai, James Alexander Pearson, Yangyang Li, Chen Chao, Lucy Zhang, Jian Peng, Yanpeng Xing, Luyao Zhang, Youjia Hu, Zhiguang Zhou, F. Susan Wong, Li Wen

**Affiliations:** ^1^National Clinical Research Center for Metabolic Diseases, Key Laboratory of Diabetes Immunology (Central South University), Ministry of Education, and Department of Metabolism and Endocrinology, The Second Xiangya Hospital, Central South University, Changsha, China; ^2^Section of Endocrinology, Department of Internal Medicine, School of Medicine, Yale University, New Haven, CT, United States; ^3^Department of Endocrinology and Metabolism, Shanghai Jiaotong University Affiliated Sixth People’s Hospital, Shanghai, China; ^4^Division of Infection and Immunity, School of Medicine, Cardiff University, Cardiff, United Kingdom; ^5^Department of Endocrinology, The Second Hospital of Jilin University, Changchun, China; ^6^Department of Gastrointestinal Surgery, The First Hospital of Jilin University, Changchun, China

**Keywords:** type 1 diabetes, interleukin-10, neutrophils, gut microbiota, CD4^+^ T cells

## Abstract

Type 1 diabetes is an autoimmune disease caused by T cell-mediated destruction of insulin-producing β cells. *BDC2.5* T cells in *BDC2.5* CD4^+^ T cell receptor transgenic Non-Obese Diabetic (NOD) mice (*BDC2.5* ^+^ NOD mice) can abruptly invade the pancreatic islets resulting in severe insulitis that progresses rapidly but rarely leads to spontaneous diabetes. This prevention of diabetes is mediated by T regulatory (Treg) cells in these mice. In this study, we investigated the role of interleukin 10 (IL-10) in the inhibition of diabetes in *BDC2.5* ^+^ NOD mice by generating *Il-10*-deficient *BDC2.5* ^+^ NOD mice (*BDC2.5* ^+^ *Il-10* ^-/-^ NOD mice). Our results showed that *BDC2.5* ^+^ *Il-10* ^-/-^ NOD mice displayed robust and accelerated diabetes development. *Il-10* deficiency in *BDC2.5* ^+^ NOD mice promoted the generation of neutrophils in the bone marrow and increased the proportions of neutrophils in the periphery (blood, spleen, and islets), accompanied by altered intestinal immunity and gut microbiota composition. *In vitro* studies showed that the gut microbiota from *BDC2.5* ^+^ *Il-10* ^-/-^ NOD mice can expand neutrophil populations. Moreover, *in vivo* studies demonstrated that the depletion of endogenous gut microbiota by antibiotic treatment decreased the proportion of neutrophils. Although *Il-10* deficiency in *BDC2.5* ^+^ NOD mice had no obvious effects on the proportion and function of Treg cells, it affected the immune response and activation of CD4^+^ T cells. Moreover, the pathogenicity of CD4^+^ T cells was much increased, and this significantly accelerated the development of diabetes when these CD4^+^ T cells were transferred into immune-deficient NOD mice. Our study provides novel insights into the role of IL-10 in the modulation of neutrophils and CD4^+^ T cells in *BDC2.5* ^+^ NOD mice, and suggests important crosstalk between gut microbiota and neutrophils in type 1 diabetes development.

## Introduction

Type 1 diabetes (T1D) is an autoimmune disease that results from the destruction of insulin-producing β cells. T cells are the predominant component of the infiltrates in these pancreatic islet lesions (1, 2). Reports from bone marrow transplantation studies in identical twins in humans (3) and adoptive transfer of T cells from diabetic Non-Obese Diabetic (NOD) mouse donors to non-diabetic NOD mouse recipients (4) demonstrated that type 1 diabetes is an immune cell-mediated disease. T cell receptor (TCR) transgenic NOD mice provide critical tools for investigation of the important roles that T cells play in the immunopathogenesis of T1D development.

The *BDC2.5* TCR transgenic NOD (*BDC2.5* ^+^ NOD) mouse was generated from a diabetogenic CD4^+^ T cell clone, designated as *BDC2.5*, from a new-onset diabetic NOD mouse (5). In these mice, ∼90% CD4^+^ T cells express the transgenic TCR (6). Interestingly, despite the potent ability of the parental *BDC2.5* T clone in inducing type 1 diabetes development, *BDC2.5* ^+^ NOD mice showed severe insulitis as young as 2-3 weeks of age but rarely developed diabetes (7, 8). However, *BDC2.5^+^* CD4^+^ T cells from the *BDC2.5* ^+^ NOD mouse could rapidly transfer diabetes into severe combined immune-deficient NOD (NOD.*scid*) recipients after activation *in vitro (* 9). Moreover, if *BDC2.5* ^+^ NOD mice are on an immunodeficient background, such as a recombination-activating gene (*Rag*) deficiency (10) or NOD.*scid* (9), the mice develop rapid diabetes at a very early age, suggesting intrinsic immune regulatory factors suppress the development of diabetes in *BDC2.5* ^+^ NOD mice in immuno-sufficient hosts. Studies have shown that Foxp3^+^CD4^+^ regulatory T (Treg) cells play a protective role by regulating autoimmune responses in *BDC2.5* ^+^ NOD mice (11–13). Our previous studies found that B cell depletion in *BDC2.5* ^+^ NOD mice induced transient aggressive behavior in diabetogenic *BDC2.5* ^+^ CD4^+^ T cells with reduction in Treg cell number and Treg cell suppressive functions (14). However, after B cell reconstitution, *BDC2.5* ^+^ CD4^+^ T cells were less aggressively pathogenic due to the increased number of Treg cells and enhanced suppressive function of Tregs cells to CD4^+^CD25^-^ T effector cells, as well as increasing IL-10 producing Bregs (14).

Although the destruction of insulin-producing pancreatic beta cells seen in type 1 diabetes is primarily characterized by autoreactive T cells, recent studies suggest that neutrophils also play an essential role in the development of type 1 diabetes. Diana et al. found that neutrophils infiltrate the islets of NOD mice at an early age, which was required for the initiation of the diabetogenic T cell response (15). The authors also showed that neutrophils interacted with B‐1a cells and plasmacytoid dendritic cells in the development of type 1 diabetes (15). Their further study showed that macrophages and β‐cells in the pancreas were responsible for neutrophil recruitment to the pancreas (16). In patients with type 1 diabetes, the level of circulating neutrophil elastase released from activated neutrophils was positively associated with the numbers and titers of the autoantibodies against β‐cell‐specific antigens, suggesting that neutrophil activation leading to the elevated proteases might be involved in the process of β‐cell autoimmunity (17). Moreover, the changes in circulating neutrophil numbers were found to be associated with β‐cell specific autoimmunity and the HLA-DR3-DQ2/DR4-DQ8 high risk genotype (18–22). Studies have shown that gut microbiota also regulated neutrophil aging and homeostasis (23, 24) and increasing evidence suggests that gut microbiota play an important role in modulating type 1 diabetes development in NOD mice (25–29) and in patients with type 1 diabetes (30, 31). It is known that IL-10 is an important immuno-regulatory cytokine in homeostasis of gut mucosal immunity (32–34) and IL-10 also mediates immune supression by Treg cells (35–37). However, it is not clear how IL-10 mediates the immune regulation seen in *BDC2.5* ^+^ NOD mice and if gut microbiota also modulate islet β cell autoimmunity through regulation of neutrophil homeostasis in *BDC2.5* ^+^ NOD mice. To fill those knowledge gaps, we generated *BDC2.5* ^+^ NOD mice with *Il-10* deficiency (*BDC2.5* ^+^ *Il-10* ^-/-^ NOD mice) and investigated the action of IL-10 in modulating diabetogenic CD4^+^ T cells, neutrophils and the gut microbiota in type 1 diabetes development.

## Methods and Materials

### Mice

Mice used in this study were housed in specific pathogen-free (SPF) facilities with a 12-hour-dark/light cycle at Yale University. NOD mice, *BDC2.5* ^+^ NOD mice, *Il-10* ^-/-^ NOD mice and NOD.*scid* mice were originally obtained from the Jackson Laboratory and maintained at Yale University. *BDC2.5* ^+^ *Il-10* ^+/+^ NOD mice and *BDC2.5* ^+^ *Il-10* ^-/-^ NOD mice were generated by breeding *BDC2.5* ^+^ NOD mice with *Il-10* ^-/-^ NOD mice, followed by intercrossing the *BDC2.5* ^+^ *Il-10* ^+/-^ NOD mice. The use of animals in this study was approved by the Institutional Animal Care and Use Committee of Yale University (approval number 2016-07911). Except for the experiments to observe diabetes incidence and adoptive transfer experiments using splenocytes from diabetic mice, all the other experiments were performed using 4-5 week-old non-diabetic mice. The detail of the number of animals used and the number of replicates are included in the figure legends for each experiment and also see **Table S1**.

### Natural History of Diabetes Development

*BDC2.5^+^Il-10^+/+^* NOD mice and *BDC2.5* ^+^ *Il-10* ^-/-^ NOD mice (both sexes) were observed for spontaneous diabetes development by screening for glycosuria (Bayer Diastix) weekly and diabetes onset was confirmed by blood glucose ≥ 250 mg/dl (13.9 mmol/l).

### Islet and Islet-Infiltrating Immune Cell Isolation

Pancreata removed from 4-week-old *BDC2.5^+^Il-10^+/+^* NOD mice and *BDC2.5* ^+^ *Il-10* ^-/-^ NOD mice were agitated in a 37°C shaking water bath after addition of 1.5 mg/ml collagenase (Sigma) and 62.5 units/ml DNase-I (Sigma). Collagenase activity was stopped by adding complete RPMI-1640 media after digestion. Islets were hand-picked under a light microscope, and subsequently incubated in a 37°C water bath for 6 minutes in the presence of 500 μl Cell Dissociation Buffer (Gibco), for immune cell isolation. After washing, isolated immune cells were filtered and re-suspended in complete RPMI-1640 media before staining.

### Intraperitoneal Glucose Tolerance Test (IPGTT)

*BDC2.5^+^Il-10^+/+^* NOD mice and *BDC2.5* ^+^ *Il-10* ^-/-^ NOD mice were fasted overnight prior to intraperitoneal injection with 20% glucose (2 g/kg). Blood glucose was measured before glucose injection and at different time points after glucose injection.

### Cell Purification

CD4^+^ T cells, antigen-presenting cells, Treg cells and neutrophils were each purified from the splenocytes of 4-week-old *BDC2.5^+^Il-10^+/+^* NOD mice, *BDC2.5* ^+^ *Il-10* ^-/-^ NOD mice, or wild type NOD mice. Splenic CD4^+^ T cells were purified by depletion, of CD8^+^ T cells (clone T1B105), MHC class II^+^ cells (clone 10.2.16), and B cells (anti-mouse IgM and IgG), incubating the cells with monoclonal antibody (mAb) hybridoma supernatants, followed by magnetic bead separation. For splenic antigen-presenting cell (APC) isolation, anti-Thy1 (Y19) mAb hybridoma supernatant and complement was used to remove Thy1^+^ T cells. The supernatants of different mAb hybridomas were kindly provided by the late Charles Janeway (Yale University). Magnetic beads conjugated with goat anti-mouse IgG, goat anti-mouse IgM, or goat anti-rat IgG were purchased from QIAGEN. Treg cells were isolated using MojoSort™ Mouse CD4^+^ T cell Isolation Kit (BioLegend), followed by CD25 positive isolation using a PE-anti-mouse CD25 antibody (Clone, PC61, BioLegend) and MojoSort™ Mouse anti-PE Nanobeads (BioLegend). The remaining CD4^+^CD25^-^ T cells were used as effector CD4^+^ T cells. Neutrophils were isolated according to the manufacturer’s instructions using MojoSort™ Mouse Neutrophil Isolation kit (BioLegend).

### *In Vitro* Culture

Splenic CD4^+^ T cells (1 × 10^5^/well) purified from 4-week-old *BDC2.5^+^Il-10^+/+^* NOD mice and *BDC2.5* ^+^ *Il-10* ^-/-^ NOD mice were stimulated with different concentrations of mimotope peptide in the presence of mitomycin-c-treated APCs for 3 days at 37°C. CD4^+^CD25^+^ Tregs (5 × 10^4^/well) purified from 4-week-old *BDC2.5^+^Il-10^+/+^* NOD mice and *BDC2.5* ^+^ *Il-10* ^-/-^ NOD were co-cultured with effector CD4^+^ T cells (CD4^+^CD25^-^ T cells, 1 × 10^5^/well) from either *BDC2.5^+^Il-10^+/+^* NOD mice or *BDC2.5* ^+^ *Il-10* ^-/-^ NOD mice, in the presence of 5 ng/ml mimotope peptide (amino acid sequence RTRPLWVRME) and mitomycin-c-treated APCs (5 × 10^4^/well), which were purified from wild-type NOD mice. Cells were incubated for 3 days at 37°C. Purified splenic neutrophils (5 × 10^4^/well) from *BDC2.5^+^Il-10^+/+^* NOD mice and *BDC2.5* ^+^ *Il-10* ^-/-^ NOD mice were co-cultured with purified splenic CD4^+^ T cells (1 × 10^5^/well) isolated from either *BDC2.5^+^Il-10^+/+^* NOD mice or *BDC2.5* ^+^ *Il-10* ^-/-^ NOD mice in the presence of mitomycin-treated APCs (5 × 10^4^/well) from wild type NOD mice and mimotope peptide (5 ng/ml). Cell proliferation was determined by ^3^H-thymidine incorporation over 16 hours, with supernatants collected prior to ^3^H-thymidine addition.

### Adoptive Transfer Experiments

Total splenocytes (10 × 10^6^) from diabetic *BDC2.5^+^Il-10^+/+^* NOD mice and *BDC2.5* ^+^ *Il-10* ^-/-^ NOD mice were injected (*i.v.*) into irradiated 4-week-old wild-type NOD mice. Total splenocytes (10 × 10^6^), or purified splenic CD4^+^ T cells (7 × 10^6^) from non-diabetic *BDC2.5^+^Il-10^+/+^* NOD mice and *BDC2.5* ^+^ *Il-10* ^-/-^ NOD mice were transferred (*i.v.*) into 4-week-old NOD.*scid* mice. Recipients were monitored for glycosuria (Bayer Diastix) weekly and diabetes was confirmed by blood glucose ≥ 250 mg/dl (13.9 mmol/l).

### Extraction of Gut Bacterial DNA

Fecal pellets collected from 4-week-old *BDC2.5^+^Il-10^+/+^* NOD mice and *BDC2.5* ^+^ *Il-10* ^-/-^ NOD mice were resuspended in 300 μl Tris-EDTA buffer (10 mM Tris and 1 mM EDTA, pH8) containing 7.5 μl 0.5% SDS and 3 μl Proteinase K (200 μg/ml). The samples were then incubated at 37°C for 1 h and homogenized in solution, containing one volume of phenol/chloroform/isoamyl alcohol (25:24:1), 200 μl 20% SDS and 0.3 g zirconium silica beads, with a mini-bead-beater (BioSpec) for 2 minutes. Phenol/chloroform/isoamyl alcohol was then added to the samples prior to centrifugation (4**°**C, 12000 g, 15 mins), with the upper aqueous layer, containing DNA, transferred to a new tube. Bacterial DNA was subsequently precipitated with isopropanol, washed with 70% ethanol, air-dried, and resuspended in 100 μl of sterile water.

### 16S rRNA Sequencing and Data Analysis

The V4 region of the bacterial 16S rRNA gene was amplified from each DNA sample by PCR using barcoded broadly-conserved primer pairs (5’-GTGCCAGCMGCCGCGGTAA-3’) and (5’-GGACTACHVGGGTWTCTAAT-3’). The PCR products were purified using gel extraction kits (QIAGEN) with DNA concentration quantified on a Nanodrop spectrophotometer. Equimolar amounts of each sample were pooled for pyrosequencing using the Ion Torrent Personal Genome Machine (PGM) sequencing system (Life Technologies). The sequencing results were analyzed with the Quantitative Insights Into Microbial Ecology (QIIME) software package (version 1.8) and UPARSE pipeline (version 7.0). β-diversity was analyzed to compare differences between microbial communities, and the data are shown as a Principal Coordinate Analysis (PCoA). Taxonomy assignment was performed at various levels using representative sequences of each operational taxonomic unit (OTU).

### Pre- and Neo-Natal Antibiotic Treatment *In Vivo*

For depletion of endogenous commensal microbiota, *BDC2.5* ^+^ *Il-10* ^+/-^ NOD breeders were treated with an antibiotic cocktail containing 0.5 g/l vancomycin, 1 g/l ampicillin, 1 g/l metronidazole, and 1 g/l neomycin added in drinking water, from one week before delivery to 4 weeks after birth.

### Cytokine ELISA

Murine IL-17A, IL-10 and IFN-γ from the serum and gut flush from different sections of the intestine were measured using the Mouse ELISA kits (BioLegend), following the manufacturer’s instructions. Serum samples were diluted 1:100 in PBS before measurement. Gut flush was obtained by infusing 10 ml PBS to the gut lumen, after termination of the mice, and removal of the intestine. The collected fluid used for ELISA, after removing the solid material by centrifugation (12,000 g, 5 min, RT).

### Gut Permeability Assay

Four-week-old *BDC2.5^+^Il-10^+/+^* NOD mice and *BDC2.5* ^+^ *Il-10* ^-/-^ NOD mice were fasted overnight for 13 hours. Food was resupplied to the mice two hours post-gavage with 0.6 mg/g FITC-dextran (MW 3,000-5,000, Sigma). Blood samples were collected from the mice two hours after food restoration and were centrifuged (2300 g, 5 min, RT) for serum separation. Serum samples were diluted 1:1 in PBS with the FITC-dextran concentration determined using a fluorescence spectrophotometer (Perkin Elmer). Standard curves were generated using known concentrations of FITC-dextran, diluted in serum from untreated NOD mice. The concentrations in serum from FITC-dextran gavaged *BDC2.5^+^Il-10^+/+^* NOD mice and *BDC2.5* ^+^ *Il-10* ^-/-^ NOD mice were determined using linear regression.

### *In Vitro* Bacterial Stimulation

Fresh stool samples, collected from 4-week-old *BDC2.5^+^Il-10^+/+^* NOD mice and *BDC2.5* ^+^ *Il-10* ^-/-^ NOD mice, were resuspended at 1 g/mL in sterile PBS, and homogenized by vortexing vigorously for 30 secs. The samples were then centrifuged (300 g, 1 min, RT) to remove large debris and subsequently further centrifuged at 12,000 g for 5 minutes to pellet the bacteria. Bacteria from *BDC2.5^+^Il-10^+/+^* NOD mice and *BDC2.5* ^+^ *Il-10* ^-/-^ NOD mice were re-suspended in sterile PBS, heat-inactivated at 95°C for 30 min, and co-cultured overnight (10^8^ CFU) with 2 million total splenocytes or purified splenic neutrophils from *BDC2.5* ^+^ *Il-10* ^+/+^ and *BDC2.5* ^+^ *Il-10* ^-/-^ NOD mice. Stimulated splenocytes or neutrophils were further analyzed by flow cytometry and real-time qPCR.

### Real Time Quantitative PCR (qPCR)

RNA from purified neutrophils (un-stimulated or stimulated with gut microbiota) or small intestinal tissue was extracted using Trizol reagent and an RNeasy mini plus kit (QIAGEN). After quantification, RNA was used for cDNA synthesis using the iScript cDNA synthesis kit (Invitrogen). Samples were analyzed on an iCycler qPCR machine (Bio-rad). Gene expression level was determined using the 2^−ΔΔCt^ method and normalized with the housekeeping gene, *Gapdh*. Primers sequences are listed in **Table 1**. Each sample was assayed in duplicate and the experiments were repeated at least twice.

**Table 1:** Primer information.

Genes	Primers	Sequence
***Tnf-α***	Forward	CAAATGGCCTCCCTCTCAT
	Reverse	TGGGCTACAGGCTTGTCACT
***Il-1β***	Forward	TGGAGAACACCACTTGTTGCTCCA
	Reverse	AAACAGATGAAGTGCTCCTTCGAGG
***Inos***	Forward	AGATTGGAGTTCGAGACTTCTG
	Reverse	TGGCTAGTGCTTCAGACTTC
***Nos2***	Forward	CCAAGCCCTCACCTACTTCC
	Reverse	CTCTGAGGGCTGACACAAGG
***Arg1***	Forward	CTCCAAGCCAAAGTCCTTAGAG
	Reverse	AGGAGCTGTCATTAGGGACATC
***Caspase9***	Forward	TCCTGGTACATCGAGACCTTG
	Reverse	AAGTCCCTTTCGCAGAAACAG
***Zonulin1***	Forward	CACCGGAGTGATGGTTTTCT
	Reverse	CCACCTCTGTCCAGCTCTTC
***Reg3β***	Forward	CTGCCTTAGACCGTGCTTTC
	Reverse	CCCTTGTCCATGATGCTCTT
***Reg3γ***	Forward	TTCCTGTCCTCCATGATCAAAA
	Reverse	CATCCACCTCTGTTGGGTTCA
***Defcr6***	Forward	CAGGCTGTGTCTGTCTCTTTTG
	Reverse	TAAATGACCCTTTCTGCAGGTC
***Gapdh***	Forward	GGGGTCGTTGATGGCAACA
	Reverse	TGTAGACCATGTAGTTGAGGTCA

### Flow Cytometry

0.5 - 1 × 10^6^ single-cell suspensions from different lymphoid tissues were incubated with Fc block at room temperature for 20 min, before cell surface staining. For intracellular cytokine staining, cells were incubated at 37°C for 4 h in the presence of 10 ng/ml phorbol myristate acetate (PMA, Sigma), 500 ng/ml of ionomycin (Sigma) and 1 μl of Golgi plug™ (BD Bioscience), followed by cell surface staining, washing, fixation and permeabilization and intracellular cytokine staining. For intranuclear staining, cells were fixed and permeabilized using the Transcription Factor Staining Buffer Set (Tonbo Biosciences). After incubation, the cells were stained with an anti-Foxp3 antibody (clone: FJK-16s, eBioscience) and T-bet (clone:4B10, BioLegend). Cells were stained with mAbs to the following surface markers: CD45 (clone: 30-F11), TCR-β (clone: H57-597), CD4 (clone: GK1.5), CD8 (clone: 53-6.7), CD11b (clone: M1/70), CD62L (clone: MEL-14), ICOS (clone: 15F9), CD69 (clone: H1.2F3), CD25 (clone: 3C7), CD19 (clone: 6D5), all from BioLegend, and Ly6G (clone: 1A8) from BD Biosciences. mAbs to intracellular cytokines include TNF-α (clone: MP6-XT22), IFN-γ (clone: XMG1.2) and IL-17A (clone: TC11-18H10.1) were purchased from Biolegend. Samples were analyzed on a BD LSRII flow cytometer and subsequently analyzed by FlowJo 8.8.6 software (Tree star). Immune cells were gated based on their FSC-A/SSC-A properties. Single cells and subsequent live cells were gated on their FSC-A/FSC-H properties and live/dead staining, respectively.

### Statistics

Diabetes incidence was compared using a log-rank test for survival. Insulitis scores were analyzed using a *Chi-*square test. Statistical analysis of microbial β-diversity was conducted using an analysis of similarities (ANOSIM). Differences between microbial species were determined following analysis using multiple *t*-tests with Bonferroni correction. Data from experiments *in vitro* were assessed for normality and subsequently analyzed using a two-tailed Student’s *t* test (if data were normally distributed), a two-tailed Mann-Whitney test (if data were not normally distributed), or a two-way ANOVA. *P* < 0.05 was considered statistically significant.

## Results

### In the Absence of IL-10, BDC2.5^+^ NOD Mice Develop Accelerated Diabetes at a Very Young Age

**Figure 1:**
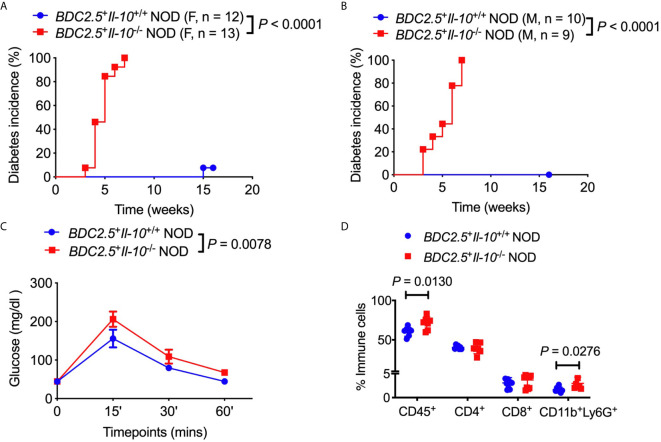
*Il-10* deficiency in *BDC2.5* ^+^ NOD mice induced rapid development of type 1 diabetes. **(A, B)** Natural history of type 1 diabetes development in *BDC2.5* ^+^ *Il-10* ^+/+^ NOD mice and *BDC2.5* ^+^ *Il-10* ^-/-^ NOD littermates from females [**(A)**, n = 12-13/group] and males [**(B)**, n = 9-10/group]. **(C)** Intraperitoneal glucose tolerance test (IPGTT) in *BDC2.5* ^+^ *Il-10* ^+/+^ NOD mice and *BDC2.5* ^+^ *Il-10* ^-/-^ NOD mice (n = 5/group). **(D)** Infiltrated immune cells in the islets of mice (*n* = 7-11/group). In flow cytometric analysis, CD45^+^ immune cells were gated from live cells. CD4^+^ T cells, CD8^+^ T cells, CD11b^+^Ly6G^+^ neutrophils were gated from CD45^+^ immune cells. Data in **(A, B, D)** were pooled from two or more independent experiments. The experiment in **(C)** was performed twice, and consistent results were obtained. Data were analyzed using a log-rank test for survival **(A, B)**, a two-way ANOVA **(C)**, or a two-tailed Student’s *t*-test [**(D)**, Data are presented as mean ± SD]. *P* < 0.05 was considered statistically significant.

To assess the role of IL-10 in diabetes protection observed in *BDC2.5^+^* NOD mice, we first monitored the natural history of type 1 diabetes development in *BDC2.5^+^Il-10^+/+^* NOD mice and *BDC2.5* ^+^ *Il-10* ^-/-^ NOD mice. Interestingly, both female and male *BDC2.5* ^+^ *Il-10* ^-/-^ NOD mice developed accelerated diabetes at a very young age (**Figures 1A, B**). In line with islet β cell destruction, *BDC2.5* ^+^ *Il-10* ^-/-^ NOD mice displayed impaired glucose tolerance compared with *BDC2.5^+^Il-10^+/+^* NOD mice (**Figure 1C**). We found no significant difference in severity of insulitis between *BDC2.5^+^Il-10^+/+^* and *BDC2.5* ^+^ *Il-10* ^-/-^ NOD mice (**Figures S1A, B**); however, *BDC2.5* ^+^ *Il-10* ^-/-^ NOD mice had more CD45^+^ immune cells, especially CD11b^+^Ly6G^+^ neutrophils, infiltrating the islets than *BDC2.5* ^+^ *Il-10* ^+/+^ NOD mice, but no significant differences in the proportion of CD4^+^ T cells, CD8^+^ T cells, B cells, macrophages, and dendritic cells (**Figure 1D** and data not shown). Our data showed that in the absence of IL-10, *BDC2.5^+^* NOD mice develop accelerated diabetes at a very young age.

### IL-10 Deficiency Promotes the Expansion of Bone Marrow and Peripheral Neutrophils in BDC2.5^+^ NOD Mice

**Figure 2:**
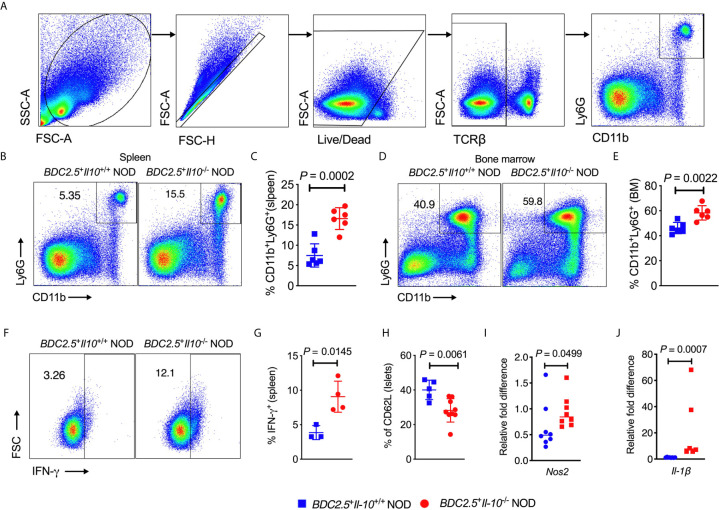
Altered neutrophil homeostasis in *BDC2.5* ^+^ *Il-10* ^-/-^ NOD mice compared with *BDC2.5* ^+^ *Il-10* ^+/+^ NOD mice. **(A)** Gating strategies of CD11b^+^Ly6G^+^ neutrophils from the spleen. **(B, C)** Proportion of CD11b^+^Ly6G^+^ neutrophils in the spleen (*n* = 6/group). Representative flow cytometric profiles **(B)**, and summary of CD11b^+^Ly6G^+^ neutrophils gated from TCR-β^-^ cells **(C)**. **(D, E)** Proportion of CD11b^+^Ly6G^+^ neutrophils in the bone marrow (*n* = 6/group). Representative flow cytometric profiles **(D)**, and summary of CD11b^+^Ly6G^+^ neutrophils gated from TCRβ^-^ cells **(E)**. **(F, G)** IFN-γ expression in splenic CD11b^+^Ly6G^+^ neutrophils (*n* = 3-4/group). Representative flow cytometric profiles **(F)**, and summary of IFN-γ expression **(G)**. **(H)** CD62L expression in pancreatic CD11b^+^Ly6G^+^ neutrophils (*n* = 5-9/group). **(I, J)** Gene expression of *Nos2* **(I)** *and Il-1β* **(J)** in splenic neutrophils from *BDC2.5* ^+^ *Il-10* ^+/+^ NOD mice and *BDC2.5* ^+^ *Il-10* ^-/-^ NOD mice (n = 6-8/group). Data in **(C, E, H–J)** were combined from two or more independent experiments. The experiment in **(G)** was performed twice and consistent results were obtained. Data in **(C, E, G, H)** are shown as mean ± SD and were analyzed using a two-tailed Student’s *t*-test. Data in **(I, J)** are presented as median and were analyzed using a two-tailed Mann-Whitney test. *P* < 0.05 was considered statistically significant.

We observed an increased frequency of neutrophils infiltrating the islets of *BDC2.5* ^+^ *Il-10* ^-/-^ NOD mice (**Figure 1D**). To investigate the crosstalk between IL-10 and neutrophils, we assessed the neutrophil population in both the bone marrow and peripheral tissues of *BDC2.5^+^Il-10^+/+^* and *BDC2.5* ^+^ *Il-10* ^-/-^ NOD mice. Consistent with the results from the islets, we also found a higher percentage of neutrophils in the spleen, bone marrow and blood of *BDC2.5* ^+^ *Il-10* ^-/-^ NOD mice, compared to *BDC2.5^+^Il-10^+/+^* NOD mice (**Figures 2A–E** and **S2A, B**). Moreover, there was a higher proportion of IFN-γ-producing splenic neutrophils in *BDC2.5^+^Il-10^-/-^* NOD mice compared with *BDC2.5* ^+^ *Il-10* ^+/+^ NOD mice (**Figures 2F, G**). The expression levels of cytokines in the neutrophils from the blood and islets in *BDC2.5^+^Il-10^+/+^* NOD mice were the same as those in *BDC2.5^+^Il-10^-/-^* NOD mice (**Figures S3A–G**). Additionally, we found that neutrophils from the islets of the *BDC2.5^+^Il-10^+/+^* NOD mice had lower levels of CD62L (**Figure 2H**). We then purified neutrophils and found no significant differences in the gene expression of *Inos*, *Nos2*, *Arg1*, and *Caspase9* in the spleen (**Figures S4A–D**); however, we found increased *Nos2* and *Il-1β* in purified neutrophils from the bone marrow of *BDC2.5* ^+^ *Il-10* ^-/-^ NOD mice (**Figures 2I, J**), but no significant differences in the expressions of *Inos*, *Arg1*, *Caspase 9*, and *Mmp9* (**Figures S4E–H**). Next, we assessed the phagocytotic ability of neutrophils and found that neutrophils from *BDC2.5* ^+^ *Il-10* ^-/-^ NOD mice were not different from those from *BDC2.5^+^Il-10^+/+^* NOD mice (**Figures S5A, B**). Taken together, our data suggested that *Il-10* deficiency altered neutrophil homeostasis in *BDC2.5* ^+^ NOD mice.

### In the Absence of IL-10, CD4^+^ T Cells Are More Activated and Pathogenic in BDC2.5^+^ NOD Mice

**Figure 3:**
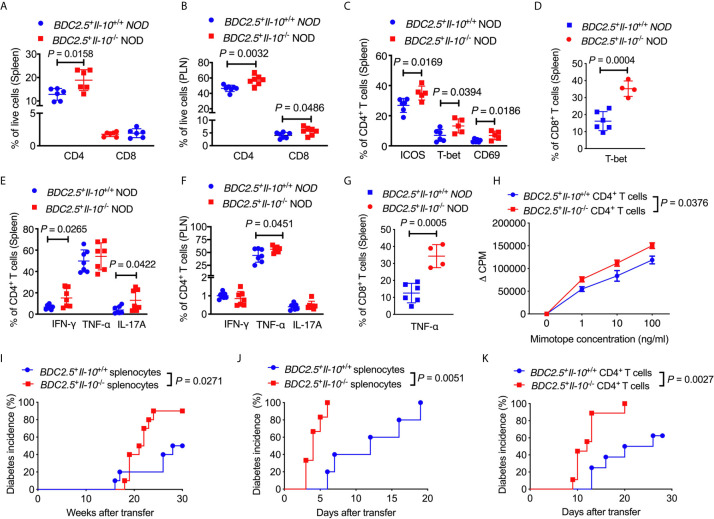
*BDC2.5*^+^*Il-10*^-/-^ NOD mice have more activated and pathogenic CD4^+^ T cells. **(A)** The percentage of CD4^+^ T cells and CD8^+^ T cells in the spleen of *BDC2.5* ^+^ *Il-10* ^+/+^ NOD mice and *BDC2.5* ^+^ *Il-10* ^-/-^ NOD mice (n = 6/group). **(B)** The percentage of CD4^+^ T cells and CD8^+^ T cells in the pancreatic lymph node (PLN) of *BDC2.5* ^+^ *Il-10* ^+/+^ NOD mice and *BDC2.5* ^+^ *Il-10* ^-/-^ NOD mice (n = 7/group). **(C)** The proportion of ICOS^+^, T-bet^+^ and CD69^+^ splenic CD4^+^ T cells in *BDC2.5* ^+^ *Il-10* ^+/+^ NOD mice and *BDC2.5* ^+^ *Il-10* ^-/-^ NOD mice (n = 5-6/group). **(D)** The proportion of T-bet^+^ splenic CD8^+^ T cells in *BDC2.5* ^+^ *Il-10* ^+/+^ NOD mice and *BDC2.5* ^+^ *Il-10* ^-/-^ NOD mice (n = 4-6/group). **(E)** The proportion of IFN-γ^+^, TNF-α^+^, and IL-17A^+^ splenic CD4^+^ T cells in *BDC2.5* ^+^ *Il-10* ^+/+^ NOD mice and *BDC2.5* ^+^ *Il-10* ^-/-^ NOD mice (n = 7/group). **(F)** The proportion of IFN-γ^+^, TNF-α^+^, and IL-17A^+^ CD4^+^ T cells in the PLN from *BDC2.5* ^+^ *Il-10* ^+/+^ NOD mice and *BDC2.5* ^+^ *Il-10* ^-/-^ NOD mice (n = 7/group). **(G)** The proportion of TNF-α^+^ splenic CD8^+^ T cells in *BDC2.5* ^+^ *Il-10* ^+/+^ NOD mice and *BDC2.5* ^+^ *Il-10* ^-/-^ NOD mice (n = 4-6/group). **(H)** Proliferation of purified splenic CD4^+^ T cells from *BDC2.5* ^+^ *Il-10* ^+/+^ NOD mice and *BDC2.5* ^+^ *Il-10* ^-/-^ NOD mice in the presence of different concentrations of mimotope. **(I)** Adoptive transfer of diabetic splenocytes from *BDC2.5* ^+^ *Il-10* ^+/+^ NOD mice and *BDC2.5* ^+^ *Il-10* ^-/-^ NOD mice into irradiated wild-type NOD mice (n = 10/group). **(J)** Adoptive transfer of non-diabetic splenocytes from *BDC2.5* ^+^ *Il-10* ^+/+^ NOD mice and *BDC2.5* ^+^ *Il-10* ^-/-^ NOD mice into NOD-*scid* mice (n = 7-9/group). **(K)** Adoptive transfer of purified splenic CD4^+^ into NOD-*scid* mice (n = 8-9/group). Data in **(A–C, E, F, I–K)** were combined from two or more independent experiments. The experiments in **(D, G, H)** were performed two or more times, with similar results obtained. Data in **(A–G)** are shown as mean ± SD. Data were analyzed using a two-tailed Student’s *t*-test **(A–G)**, a two-way ANOVA **(H)**, or log-rank test for survival **(I–K)**. *P* < 0.05 was considered statistically significant.

Next, we assessed the phenotype and function of different T cell populations in *BDC2.5* ^+^ *Il-10* ^-/-^ NOD mice. Interestingly, unlike the composition of the immune cells seen in the islet infiltrate (**Figure 1D**), *BDC2.5* ^+^ *Il-10* ^-/-^ NOD mice had a higher proportion of splenic CD4^+^ T cells when compared with *BDC2.5^+^Il-10^+/+^* NOD mice (**Figure 3A**), while no significant difference was found in the percentage of CD8^+^ T cells (**Figure 3A**). The proportions of both CD4^+^ and CD8^+^ T cells were increased in the pancreatic lymph node (PLN) of *BDC2.5* ^+^ *Il-10* ^-/-^ NOD mice (**Figure 3B**). We found that splenic CD4^+^ T cells from *BDC2.5* ^+^ *Il-10* ^-/-^ mice expressed a higher proportion of ICOS, T-bet and CD69 (**Figure 3C**) while splenic CD8^+^ T cells expressed higher level of T-bet only (**Figure 3D**). Moreover, splenic CD4^+^ T cells from *BDC2.5* ^+^ *Il-10* ^-/-^ mice showed increased expressions of inflammatory cytokines including IFN-γ and IL-17A in the spleen (**Figure 3E**) and more TNF-α in CD4^+^ T cells from PLN (**Figure 3F**). Additionally, splenic CD8^+^ T cells in *BDC2.5* ^+^ *Il-10* ^-/-^ NOD mice expressed more TNF-α (**Figure 3G**). Interestingly, splenic CD4^+^ T cells from *BDC2.5* ^+^ *Il-10* ^-/-^ NOD mice showed stronger proliferative responses to their antigenic peptide (mimotope) compared to CD4^+^ T cells from *BDC2.5^+^Il-10^+/+^* splenocytes (**Figure 3H**). No significant differences were found in the proportion of CD8^+^ T cells expressing IFN-γ, TNF-α, or IL-17A in the PLN between *BDC2.5* ^+^ *Il-10^+/+^* and *BDC2.5^+^Il-10* ^-/-^ NOD mice (**Figures S6A–C**). To determine the effect of IL-10 on diabetogenicity of the T cells from *BDC2.5^+^Il-10* ^-/-^ NOD mice *in vivo*, we adoptively transferred total splenocytes from diabetic *BDC2.5^+^Il-10* ^-/-^ or *BDC2.5* ^+^ *Il-10^+/+^* NOD mice into 4-week-old irradiated wild-type female NOD mice. Supporting the *in vitro* data, splenocytes from diabetic *BDC2.5* ^+^ *Il-10* ^-/-^ NOD mice showed more potent diabetogenicity by inducing a higher incidence of diabetes in the recipients, compared with the splenocytes from *BDC2.5^+^Il-10^+/+^* NOD mice (**Figure 3I**). Moreover, when we transferred total splenocytes from non-diabetic *BDC2.5* ^+^ *Il-10* ^-/-^ or *BDC2.5^+^Il-10^+/+^* NOD mice into NOD.*scid* mice, splenocytes from *BDC2.5* ^+^ *Il-10* ^-/-^ mice induced rapid diabetes in the NOD.*scid* recipients as early as 3-days after transfer (**Figure 3J**). We further confirmed the enhanced diabetogenicity by BDC2.5 CD4^+^ T cells by adoptive transfer with purified splenic CD4^+^ T cells from non-diabetic *BDC2.5* ^+^ *Il-10* ^-/-^ or *BDC2.5^+^Il-10^+/+^* NOD mice into NOD.*scid* recipients. Similar to the adoptive transfer with total splenocytes, CD4^+^ T cells from *BDC2.5* ^+^ *Il-10* ^-/-^ NOD mice induced rapid onset of diabetes compared to their counterparts from *BDC2.5^+^Il-10^+/+^* NOD mice (**Figure 3K**). To determine the effects of neutrophils on CD4^+^ T cells, we performed criss-cross co-culture in which purified splenic neutrophils from *BDC2.5* ^+^ *Il-10* ^+/+^ *or BDC2.5* ^+^ *Il-10* ^-/-^ NOD mice were cultured with purified splenic CD4^+^ T cells from *BDC2.5* ^+^ *Il-10* ^-/-^ or *BDC2.5* ^+^ *Il-10* ^+/+^ NOD mice. Interestingly, neutrophils from both *BDC2.5* ^+^ *Il-10* ^+/+^ and *BDC2.5* ^+^ *Il-10* ^-/-^ NOD mice effectively inhibited the proliferation of CD4^+^ T cells from *BDC2.5* ^+^ *Il-10* ^+/+^ and *BDC2.5* ^+^ *Il-10* ^-/-^ NOD mice when stimulated by antigenic peptide (**Figures S7A, B**). However, there was no significant difference in the inhibitory effect of neutrophils from *BDC2.5* ^+^ *Il-10* ^+/+^ and *BDC2.5* ^+^ *Il-10* ^-/-^ NOD mice (**Figures S7A, B**). When we transferred purified splenic CD4^+^ T cells alone or purified splenic neutrophils together with CD4^+^ T cells from *BDC2.5* ^+^ *Il-10* ^-/-^ NOD mice into *Rag* ^-/-^ NOD mice, no significant difference in the diabetes incidence was found, indicating that the altered neutrophils in *BDC2.5* ^+^ *Il-10* ^-/-^ NOD mice may contribute to the disease development, but not by direct effects on CD4^+^ T cells (**Figure S7C**). We examined the Treg cells in *BDC2.5* ^+^ *Il-10* ^-/-^ NOD mice. Surprisingly, there were no significant differences in both the proportion and the suppressive function of Treg cells between *BDC2.5^+^Il-10^+/+^* and *BDC2.5* ^+^ *Il-10* ^-/-^ NOD mice (**Figures S8A–C**). Taken together, our data showed that in the absence of *Il-10*, CD4^+^ T cells in *BDC2.5* ^+^ NOD mice were more activated and highly pathogenic. However, Tregs did not appear to be directly affected by the absence of *Il-10*.

### IL-10 Deficiency Alters the Composition of Gut Bacteria and Intestinal Immunity in BDC2.5^+^ NOD Mice

**Figure 4:**
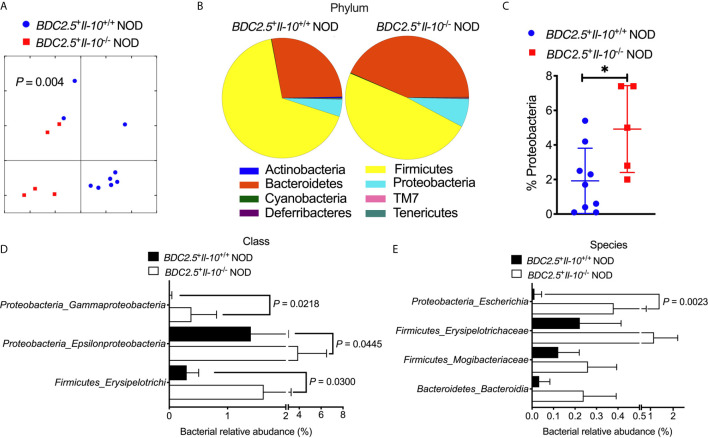
Gut bacterial composition of *BDC2.5* ^+^ *Il-10* ^-/-^ NOD mice showed distinct differences compared with *BDC2.5* ^+^ *Il-10* ^-/-^ NOD mice. Gut bacteria in fecal pellets were analyzed by 16S rRNA sequencing (*n* = 5-9/group). **(A)** β-diversity of the gut bacteria indicated by unweighted PCoA. **(B, C)** Gut bacterial composition at the phylum level including representative composition **(B)** and summarized percentage of *Proteobacteria* as an example **(C)**. **(D)** Gut bacterial composition at the class level. **(E)** Gut bacterial composition at the species level. Statistical analysis was performed by an analysis of similarities (ANOSIM) **(A)**, a two-tailed Student’s *t*-test **(C)**, or multiple *t*-tests with Bonferroni correction **(D, E)**. *P* < 0.05 was considered statistically significant. *P = 0.0261.

**Figure 5:**
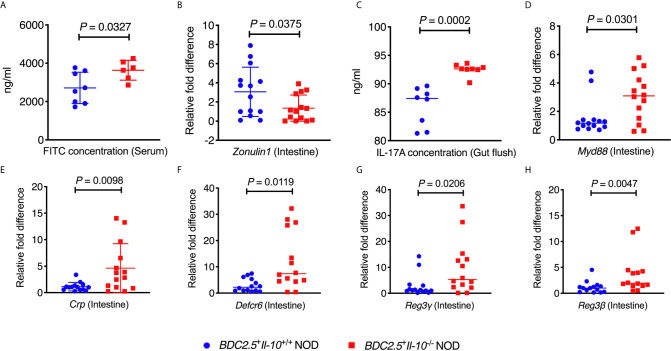
*BDC2.5*^+^*Il-10*^-/-^ NOD mice showed altered intestinal responses compared with *BDC2.5* ^+^ *Il-10* ^+/+^ NOD mice. **(A)** Gut permeability assessed following FITC-dextran uptake into the blood from the gut (*n* = 6-8/group). **(B)** Gene expression of *Zonulin1* (*n* = 14/group). **(C)** IL-17A concentration in the gut flush of the large intestine (*n* = 8/group). **(D)** Gene expression of *Myd88* (*n* = 14/group). **(E–H)** Gene expression of intestinal antimicrobial peptide genes, including *Crp* **(E)**, *Defcr6* **(F)**, *Reg3γ* **(G)**, and *Reg3β* **(H)** (*n* = 14/group). Data in **(A–H)** were combined from two or more independent experiments are presented as mean ± SD **(A, B, E)** or Median **(C, D, F–H)**. Statistical analysis was performed by a two-tailed Student’s *t*-test **(A, B, E)** or a two-tailed Mann-Whitney test **(C, D, F–H)**. *P* < 0.05 was considered statistically significant.

To study whether *Il-10* deficiency also alters the gut bacteria of TCR transgenic *BDC2.5* ^+^ NOD hosts, we investigated the gut bacterial composition of *BDC2.5* ^+^ *Il-10* ^+/+^ and *BDC2.5* ^+^ *Il-10* ^-/-^ NOD mice. *Il-10*-deficient *BDC2.5* ^+^ NOD mice had an altered composition of gut bacteria, indicated by β-diversity of the gut microbiota shown in the Principal Coordinate Analysis (PCOA; **Figure 4A**), compared to *Il-10*-sufficient *BDC2.5* ^+^ NOD mice. Further analysis also revealed differences at the phylum, class, and species levels. At the phylum level, *BDC2.5* ^+^ *Il-10* ^-/-^ NOD mice had increased levels of *Proteobacteria* compared with *BDC2.5* ^+^ *Il-10* ^+/+^ NOD mice (**Figures 4B, C**), with increased abundance of *Epsilonproteobacteria* and *Gammaproteobacteria* (**Figure 4D**). Moreover, the relative abundance of *Escherichia* from *Proteobacteria* was significantly different between the two groups (**Figure 4E**). We also assessed gut permeability *in vivo* to determine whether the altered gut bacteria affected the gut barrier in *Il-10*-deficient *BDC2.5* ^+^ NOD mice. Indeed, *BDC2.5* ^+^ *Il-10* ^-/-^ NOD mice exhibited increased gut permeability compared to *BDC2.5* ^+^ *Il-10* ^+/+^ NOD mice as demonstrated by the increased concentration of serum FITC-dextran (**Figure 5A**). We found decreased expression of the intestinal tight junction protein *Zonulin1* in *BDC2.5* ^+^ *Il-10* ^-/-^ NOD mice (**Figure 5B**), but not *Claudin2* or *Occludin* (data not shown). Moreover, when we assessed the inflammatory cytokines in the gut lumen, we found an increased level of IL-17A (**Figure 5C**) in *BDC2.5* ^+^ *Il-10* ^-/-^ NOD mice. Additionally, the intestinal expression of *Myd88*, which plays an essential role in the activation of innate immunity, was also increased in *BDC2.5* ^+^ *Il-10* ^-/-^ NOD mice when compared with that in *BDC2.5* ^+^ *Il-10* ^+/+^ NOD mice (**Figure 5D**). Interestingly, *BDC2.5* ^+^ *Il-10* ^-/-^ NOD mice showed increased expression of intestinal antimicrobial peptide genes, including *Crp*, *Defcr6*, *Reg3γ*, and *Reg3β* (**Figures 5E–H**), which could be induced to defend against the altered bacteria in the gut. Additionally, we found that the percentages of TCR-γδ^+^ cells and NKP46^+^ (one of the ILC markers) cells decreased in *BDC2.5* ^+^ *Il-10* ^-/-^ NOD mice, but no significant differences in CD11c^+^ cells, IFN-γ^+^CD4^+^ (Th1) cells, IL-17^+^CD4^+^ (Th17) cells, and CD117^+^ (one of the ILC markers) cells between the two groups (**Figures S9A–F**). Taken together, *Il-10* deficiency in *BDC2.5* ^+^ NOD mice not only altered the gut bacterial composition, but also changed the intestinal immune responses of the host.

### Altered Gut Bacteria Contribute to the Altered Neutrophil Homeostasis in *BDC2.5^+^Il-10^-/-^*s NOD Mice

**Figure 6:**
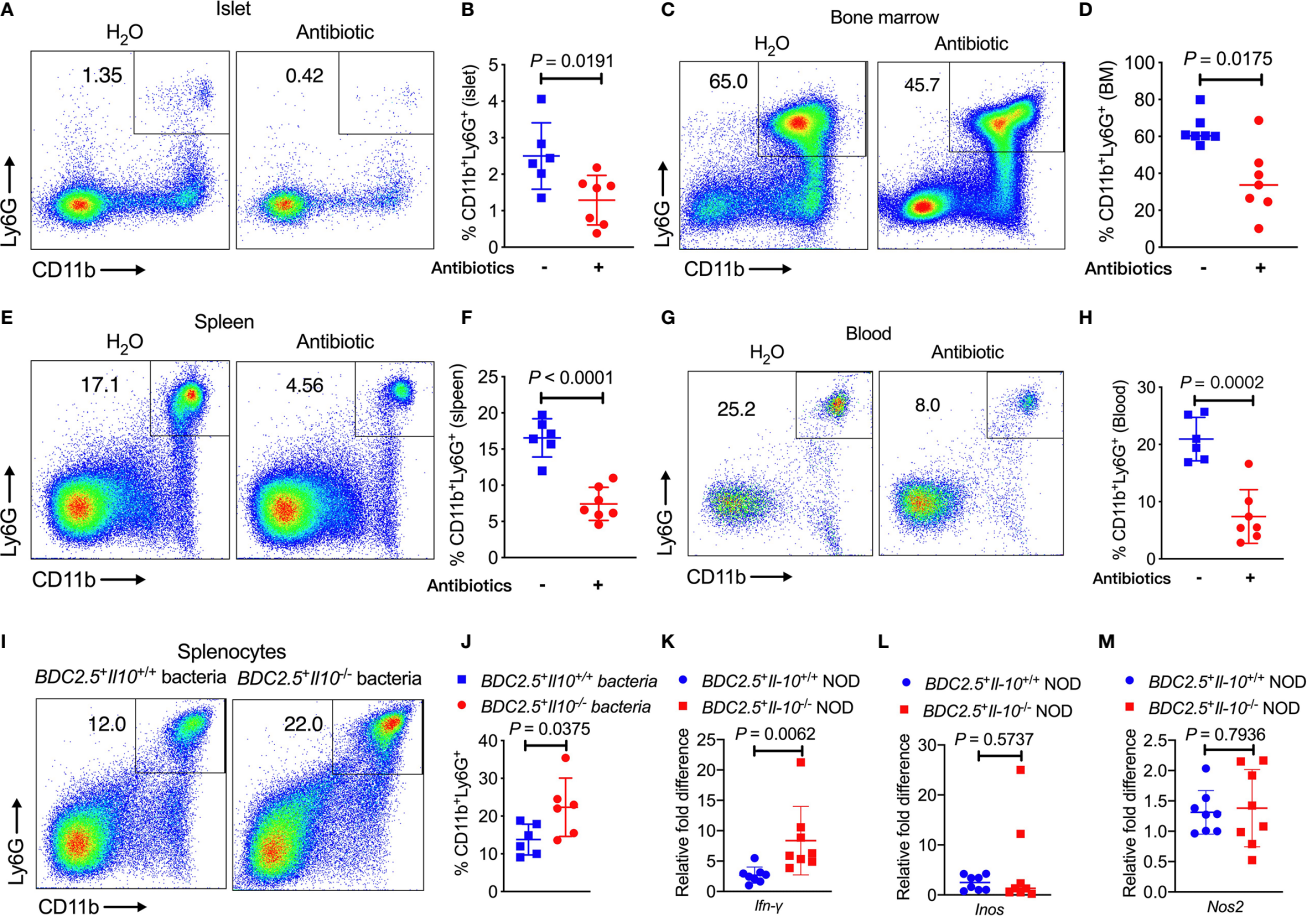
*BDC2.5*^+^*Il-10*^-/-^ NOD gut bacteria modulate neutrophil homeostasis. **(A–H)** *BDC2.5* ^+^ *Il-10* ^-/-^ NOD mice showed altered percentages of neutrophils after antibiotic treatment (*n* = 6-7/group). Representative flow cytometric profiles, and summary of CD11b^+^Ly6G^+^ neutrophils in the islets gated from CD45^+^ immune cells **(A, B)**, in the bone marrow gated from TCRβ^-^ cells **(C, D)**, in the spleen gated from TCRβ^-^ cells **(E, F)**, and in the blood gated from live cells **(G, H)**. **(I, J)** Proportion of CD11b^+^Ly6G^+^ neutrophils in the splenocytes stimulated with heat-inactivated gut bacteria from either *BDC2.5* ^+^ *Il-10* ^+/+^ NOD mice or *BDC2.5* ^+^ *Il-10* ^-/-^ NOD mice (*n* = 6/group). Representative flow cytometric profiles **(I)**, and summary of CD11b^+^Ly6G^+^ neutrophils gated from TCR-β^-^ cells **(J)**. **(K–M)** Gene expression of *Ifn-γ* **(K)** *, Inos* **(L)**, and *Nos2* **(M)** from purified splenic neutrophils after stimulation with heat-inactivated gut bacteria from either *BDC2.5* ^+^ *Il-10* ^+/+^ NOD mice or *BDC2.5* ^+^ *Il-10* ^-/-^ NOD mice (*n* = 8/group). Data in **(B, D, F, H, J–M)** were combined from two or more independent experiments. Data in **(B, F, H, J, K, M)** are presented as mean ± SD and statistical analysis was performed by a two-tailed Student’s *t*-test. Data in **(D, L)** are presented as Median and were analyzed using a two-tailed Mann-Whitney test. *P* < 0.05 was considered statistically significant.

To determine whether the altered gut bacteria in *BDC2.5* ^+^ *Il-10* ^-/-^ NOD mice contribute to the increased frequency of neutrophils, we first depleted the endogenous commensal bacteria of *BDC2.5* ^+^ *Il-10* ^+/-^ NOD breeders by treating the mice with a cocktail of antibiotics (0.5 g/L vancomycin, 1 g/L ampicillin, 1 g/L metronidazole, and 1 g/L neomycin) in drinking water and investigated the changes in neutrophils. Our results revealed that the antibiotic treatment significantly decreased the proportions of neutrophils in the islet, bone marrow, spleen and peripheral blood (**Figures 6A–H**). We also had the wildtype mice (*BDC2.5* ^+^ *Il-10* ^+/+^) as controls, treated with or without antibiotic, and found that neutrophils were only decreased in the spleen in *BDC2.5* ^+^ *Il-10* ^+/+^ NOD mice (**Figure S10**). To further confirm the role of gut microbiota from *BDC2.5* ^+^ *Il-10* ^-/-^ NOD mice in modulating neutrophil homeostasis, we co-cultured *BDC2.5* ^+^ *Il-10* ^-/-^ NOD splenocytes *in vitro* with heat-inactivated gut bacteria from either *BDC2.5* ^+^ *Il-10* ^+/+^ or *BDC2.5* ^+^ *Il-10* ^-/-^ NOD mice. Interestingly, we found that gut microbiota from *BDC2.5* ^+^ *Il-10* ^-/-^ NOD further expanded the neutrophil population significantly (**Figures 6I, J**). Next, we purified splenic neutrophils from *BDC2.5* ^+^ *Il-10* ^-/-^ NOD mice and stimulated them with heat-inactivated gut bacteria from either *BDC2.5* ^+^ *Il-10* ^+/+^ or *BDC2.5* ^+^ *Il-10* ^-/-^ NOD mice, followed by assessment of gene expression. Our qPCR results showed that *BDC2.5* ^+^ *Il-10* ^-/-^ NOD gut microbiota promoted *Ifn-γ* gene expression (**Figure 6K**), which was consistent with the intracellular cytokine staining results seen in *BDC2.5* ^+^ *Il-10* ^-/-^ NOD mice (**Figures 2F, G**). Similar to the results using fresh *ex-vivo* splenic neutrophils (**Figures S4A, B**), there were no significant differences in the gene expressions of *Inos* and *Nos2* in the neutrophils after *in vitro* stimulation (**Figures 6L, M**). Taken together, our data suggested that altered gut bacteria from *BDC2.5* ^+^ *Il-10* ^-/-^ NOD mice modulated neutrophil homeostasis, which play a role in the development of type 1 diabetes.

## Discussion

In this study, we developed a markedly accelerated model of type 1 diabetes by introducing an *Il-10* deficiency to *BDC2.5* ^+^ NOD mice, whereas *Il-10* sufficient *BDC2.5* ^+^ NOD mice develop a very low incidence and delayed spontaneous diabetes. Although no significant difference was found in the severity of insulitis between *BDC2.5* ^+^ *Il-10* ^+/+^ NOD mice and *BDC2.5* ^+^ *Il*-10^-/-^ NOD mice, *BDC2.5* ^+^ *Il-10* ^-/-^ NOD mice had more immune cells, especially neutrophils, infiltrating into the islets. In this mouse model, *BDC2.5* ^+^ NOD CD4^+^ T cells are more activated and pathogenic than CD4^+^ T cells from *Il-10* sufficient *BDC2.5* ^+^ NOD mice. Furthermore, *BDC2.5* ^+^ *Il-10* ^-/-^ NOD mice displayed increased proportions of neutrophils in the bone marrow, peripheral blood and spleen, which was closely associated with altered gut microbiota. We postulate that the altered microbiota may be central in increasing the neutrophils, which also play a role in increasing pathogenic CD4^+^ T cells.

IL-10 is a multifunctional cytokine that plays crucial roles in limiting the immune response and regulating the growth and/or differentiation a variety of immune cells (38, 39). The reports of the role of IL-10 in the development of type 1 diabetes are conflicting. It has been documented that early exposure to IL-10 accelerates the disease development (40), while IL-10 exposure during the later prediabetic phase inhibits disease (41). Additionally, the effects of IL-10 on autoimmune diabetes of NOD mice are associated with the location of IL-10 expression. Pancreatic expression of IL-10 can up-regulate the expression of intercellular adhesion molecule 1 (ICAM-1) on vascular endothelium (42) and promotes diabetes development (43), but systemic IL-10 is dispensable for autoimmune diabetes (44). IL-10 has the ability to drive the generation and differentiation of Treg cells that can inhibit the antigen-specific immune responses (45, 46). Moreover, IL-10 also affects the function of Treg cells (35). In NOD mice, overexpression of IL-10 can dramatically induce Treg cells and therefore ameliorates the development of type 1 diabetes (47). However, we found that *Il-10* deficiency in *BDC2.5* ^+^ NOD mice did not affect the frequency and function of Treg cells. Consistent with previous findings that IL-10 strongly inhibited the cytokine production (48) and the proliferation of CD4^+^ T cells (49), we found that *Il-10* deficiency significantly affected the cytokine expression in CD4^+^ T cells and enhanced the immune response of *BDC2.5* ^+^ CD4^+^ T cells to specific antigen stimulation. Moreover, *Il-10* deficiency modulated the activation of *BDC2.5* ^+^ NOD CD4^+^ T cells, with increased expression of T-bet, CD69, IFN-γ, TNF-α and IL-17A. Most importantly, *Il-10* deficiency changed the function of CD4^+^ T cells as *BDC2.5* ^+^ *Il-10* ^-/-^ CD4^+^ T cells were more pathogenic and induced rapid diabetes onset in NOD.*scid* mice compared with those CD4^+^ T cells from *BDC2.5* ^+^ *Il-10* ^+/+^ NOD mice. Therefore, *Il-10* deficiency in *BDC2.5* ^+^ NOD mice significantly affects the activation, proliferation, and function of CD4^+^ T cells, and thus contributes to the development of type 1 diabetes.

Although T cells have been well documented to play predominant roles in type 1 diabetes development, there is increasing evidence from both animal models and human beings that neutrophils from the innate immune system also contribute the initiation and progression of type 1 diabetes (15, 17, 21, 22). In addition to any effects on CD4^+^ T cells, *Il-10* deficiency had a significant impact on homeostasis of neutrophils in different tissues and expanded neutrophils in the bone marrow, peripheral blood, spleen, and islets in *BDC2.5* ^+^ NOD mice. Interestingly, we found that the change in the frequency of neutrophils was mediated by the altered gut microbiota in *BDC2.5* ^+^ *Il-10* ^-/-^ NOD mice, as this expansion was ameliorated by the depletion of the endogenous commensal microbiota *via* antibiotic treatment. Our work furthers the current understanding from other studies where gut microbiota was shown to regulate the host immunity by influencing neutrophil production and activation (50). Khosravi et al. also demonstrated that germ-free (GF) animals displayed reduced proportions of neutrophils in bone marrow and spleen (51). Microbially-derived components can regulate neutrophil homeostasis as the neutrophil reduction was rescued by treatment with microbe-associated molecular patterns from heat-killed *E coli* or autoclaved cecal content (51). Gut microbiota have been documented to regulate granulocytosis and neutrophil homeostasis by influencing the intestinal IL-17-producing cells and the release of granulocyte colony-stimulating factor (G-CSF) in a Toll-like receptor 4 (TLR4)/myeloid differentiation factor 88 (MyD88)-dependent manner (23). Additionally, Zhang and colleagues found that gut microbiota modulate the neutrophil ageing and the depletion of the microbiota can significantly reduce the number of circulating aged neutrophils (24). Our studies suggest that gut microbiota might affect type 1 diabetes development through modulating neutrophil hemostasis, especially by increasing neutrophil infiltration in the islets. Further investigation is needed to elucidate how gut microbiota modulate neutrophils and their role in the development of type 1 diabetes. To better understand the role of IL-10 in regulation of gut microbiota and gut microbiota-associated neutrophil hemostasis, the ideal approach would be to generate germ free *Il-10* deficient mice. This would be our future direction.

Taken together, by depleting *Il-10* in *BDC2.5* ^+^ NOD mice, we generated a markedly accelerated model of type 1 diabetes. Our studies showed that *Il-10* deficiency in *BDC2.5* ^+^ NOD mice significantly altered the immune response and function of CD4^+^ T cells. Importantly, we showed that the effect of IL-10 on the homeostasis of neutrophils was mediated by the altered gut microbiota. Thus, our study suggests that gut microbiota might contribute to the development of type 1 diabetes by regulation of neutrophil homeostasis. These findings may provide novel insights into the role of IL-10 in the modulation of both innate immune cells and adaptive immune cells and the development of autoimmunity, such as type 1 diabetes.

## Data Availability Statement

The raw data supporting the conclusions of this article will be made available by the authors, without undue reservation.

## Ethics Statement

The animal study was reviewed and approved by The Institutional Animal Care and Use Committee of Yale University.

## Author Contributions 

LW conceived the project. JH, QT, NT, JAP, YL, CC, LZ, JP, YX, LYZ, and YH researched the data. ZZ contributed to the discussion. JH wrote the manuscript. LW, FW, and JAP revised the manuscript. All authors contributed to the article and approved the submitted version.

## Funding

This work was supported by the Foundation for the National Institutes of Health (DK 045735, HD 097808, DK 126809), Diabetes Research Connection and Diabetes Action of Research and Education Foundation to LW. FW was funded MRC research grant (MR/K021141) and JAP was supported by JDRF postdoc fellowship and currently by MRC Career Development Award (MR/T10525). JH is a recipient of the Pilot & Feasibility grant of Yale Diabetes Research Center (DK 045735).

## Conflict of Interest

The authors declare that the research was conducted in the absence of any commercial or financial relationships that could be construed as a potential conflict of interest.

## Publisher’s Note

All claims expressed in this article are solely those of the authors and do not necessarily represent those of their affiliated organizations, or those of the publisher, the editors and the reviewers. Any product that may be evaluated in this article, or claim that may be made by its manufacturer, is not guaranteed or endorsed by the publisher.

## Abbreviations

FITC, fluorescein isothiocyanate; G-CSF, granulocyte colony-stimulating factor; IL-10, interleukin 10; MyD88, myeloid differentiation factor 88; NOD, non-obese diabetic; OTU, operational taxonomic unit; PBS, Phosphate Buffered Saline; PCoA, Principal Coordinate Analysis; PLN, pancreatic lymph node; Rag, recombination-activating gene; Scid, severe combined immune deficiency; SPF, specific pathogen-free; TLR4, Toll-like receptor 4.
