# Supplementary material for: IL-10 Deficiency Accelerates Type 1 Diabetes Development via Modulation of Innate and Adaptive Immune Cells and Gut Microbiota in BDC2.5 NOD Mice
Source: Front Immunol. 2021 Jul 30;12:702955. doi: 10.3389/fimmu.2021.702955 (PMC8362616; doi:10.3389/fimmu.2021.702955)
Supplement: Supplementary file 1 [file DataSheet_1.docx]

**Supplemental materials**

**Supplemental methods**

***Insulitis Score*** Pancreata were collected from 4-week-old *BDC2.5^+^Il-10^+/+^* NOD mice and *BDC2.5*^+^*Il-10*^-/-^ NOD mice, fixed in 10% neutral formalin buffer and embedded in paraffin. Paraffin-embedded pancreata were then sectioned at a thickness of 5 μm and stained with hematoxylin-eosin. Insulitis was scored under light microscopy using the following grading scale: 0, no infiltration; 1, < 25% infiltration of the islet; 2, 25% - 75% infiltration of the islet; and 3, > 75% islet infiltration.

***Neutrophil phagocytosis assay*** Fifty µl of whole blood were taken from 4-week-old *BDC2.5^+^Il-10^+/+^* NOD mice and *BDC2.5*^+^*Il-10*^-/-^ NOD mice. 1 ml fluorescein isothiocyanate (FITC)-dextran (1 mg/ml in sterile PBS) was added to the whole blood prior to incubation at 37°C for 60 minutes. The reaction was stopped by adding 1 ml of PBS. Red blood cells were subsequently lysed and the cells were stained with 2 µl of Fc block (Biolegend), prior to anti-CD11b and anti-Ly6G monoclonal antibody staining. Samples were analyzed on a BD LSR-II flow cytometer and subsequently analyzed using FlowJo 8.8.6 (Tree star).

**Supplemental results**

**
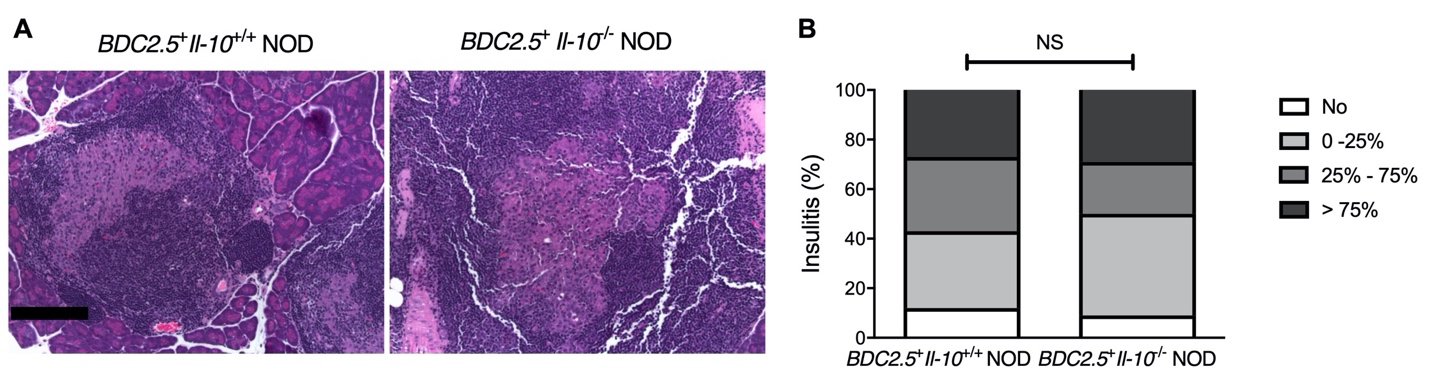
**

**Figure S1. Insulitis scores of *BDC2.5*^+^*Il-10*^+/+^ NOD mice and *BDC2.5*^+^*IL-10*^-/-^ NOD mice.** (**A-B**) Representative insulitis images of microscopic views (× 200, scale bar, 50 μm) showing differences in immune cell infiltration (A), and summarized percentages of scoring of insulitis (B). Pancreata from non-diabetic *BDC2.5*^+^*Il-10*^+/+^ NOD mice and *BDC2.5*^+^*Il-10*^-/-^ NOD mice (*n* = 6/group) were fixed in 10% neutral formalin and insulitis was evaluated under light microscope after H&E staining. At least 100 islets were examined, and insulitis was scored as shown. Data were pooled from two independent experiments and analyzed using a *Chi*-square test in (B). *P* < 0.05 was considered statistically significant.

**Figure S2.** (**A-B**) Proportion of CD11b^+^Ly6G^+^ neutrophils in the blood (*n* = 6/group). Representative flow cytometric profiles (A), and summary of CD11b^+^Ly6G^+^ neutrophils gated from live cells (B). Data are shown as mean ± SD and were analyzed using a two-tailed Student's *t*-test. *P* < 0.05 was considered statistically significant.

**Figure S3.** **Cytokines expressed in CD11b^+^Ly6G^+^ neutrophils in the blood and islets from *BDC2.5*^+^ *Il-10*^+/+^ NOD mice and *BDC2.5*^+^*Il-10*^-/-^ NOD mice.** (**A**) IFN-γ in neutrophils from the blood. (**B**) IL-17A in neutrophils from the blood. (**C**) TNF-α in neutrophils from the blood. (**D**) IFN-γ in neutrophils from the islets. (**E**) IL-17A in neutrophils from the islets. (**F**) TNF-α in neutrophils from the islets. CD11b^+^Ly6G^+^ neutrophils were gated from CD45^+^ immune cells (*n* = 4-8/group). Data are shown as mean ± SD and were analyzed using a two-tailed Student's *t*-test. *P* < 0.05 was considered statistically significant.

**Figure S4. Gene expression profile in neutrophils from the spleen and bone marrow of *BDC2.5*^+^*Il-10*^+/+^ NOD mice and *BDC2.5*^+^*Il-10*^-/-^ NOD mice.** (**A-D**) Gene expression of *Inos* (A), *Nos2* (B), *Arg1* (C) and *Caspase9* (D) in neutrophils from the spleen. (**E-H**) Gene expression of *Inos* (E), *Arg1* (F), *Caspase9* (G*),* and *Mmp9* (H) in neutrophils from the bone marrow. The experiments in (A-H) were performed twice (*n* = 6-8/group), and consistent results were obtained. Data in (A-B) are presented as mean ± SD and were analyzed by a two-tailed Student's *t*-test. Data in (C-H) are presented as median and were analyzed by a two-tailed Mann-Whitney test. *P* < 0.05 was considered statistically significant.

**
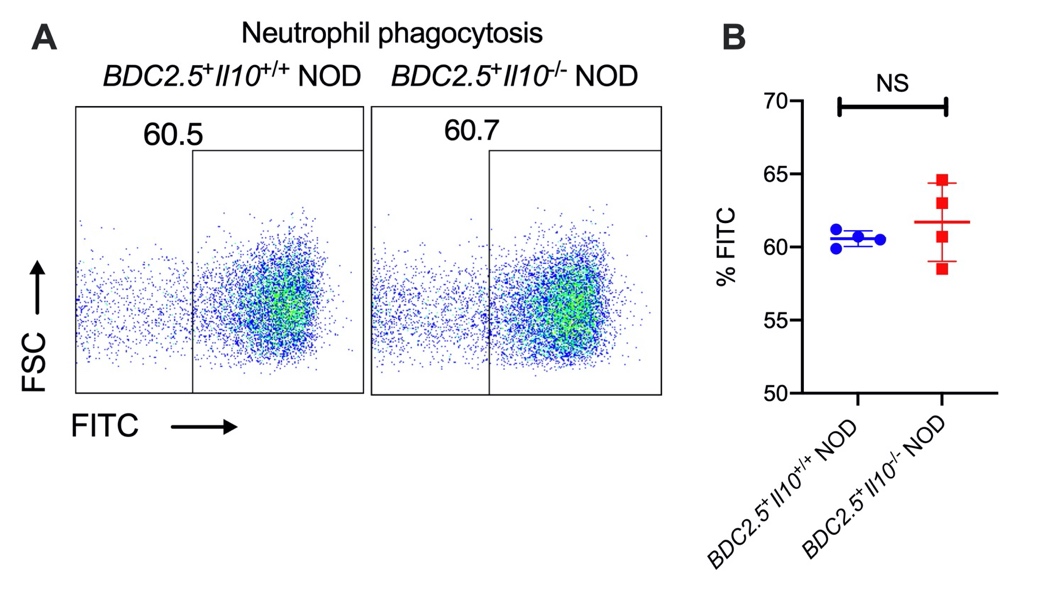
**

**Figure S5. Neutrophil phagocytotic ability.** (**A-B**) Neutrophil phagocytosis of FITC-dextran (*n* = 4/group). Representative flow cytometric profiles of FITC^+^ cells gated from CD11b^+^Ly6G^+^ cells (A), and summary of FITC^+^CD11b^+^Ly6G^+^ neutrophils (B). The experiment was performed twice, and consistent results were obtained. Data in (B) are shown as mean ± SD, and were analyzed using a two-tailed Student's *t*-test. *P* < 0.05 was considered statistically significant.

**Figure S6. Expressions of cytokines in CD8^+^ T cells in the pancreatic lymph node.** (**A-C**) The expression of cytokines in CD8^+^ T cells in the pancreatic lymph node (PLN) of *BDC2.5*^+^*Il-10*^+/+^ NOD mice and *BDC2.5*^+^*Il-10*^-/-^ NOD mice (*n* = 4-6/ group). IFN-γ (A), TNF-α (B), and IL-17A (C). The experiments in (A-C) were performed twice, and consistent results were obtained. Data were analyzed using a two-tailed Student's *t*-test and are shown as mean ± SD. *P* < 0.05 was considered statistically significant.

**Figure S7. Neutrophil suppressive function and interaction with purified *BDC2.5*^+^*Il-10*^+/+^ NOD CD4^+^ T cells.** (**A-B**) *BDC2.5*^+^*Il-10*^+/+^ NOD and *BDC2.5*^+^*Il-10*^-/-^ NOD splenic neutrophil suppressive function *in vitro* on *BDC2.5*^+^*Il-10*^+/+^ NOD CD4^+^ T cells (A) and *BDC2.5*^+^*Il-10*^-/-^ NOD CD4^+^ T cells (B). (**C**) Neutrophils function on BDC2.5 CD4^+^ T cells *in vivo* (*n* = 5-6/group). The experiments in (A-B) were performed twice, and consistent results were obtained. Data are shown as mean ± SD, and were analyzed using a one-way ANOVA test, followed by a two-tailed Student's *t*-test between groups. Data in (C) were pooled from two independent experiments and were analyzed using a log-rank test for survival. *P* < 0.05 was considered statistically significant.

**Figure S8. Treg cell proportion and function in *BDC2.5*^+^ *Il-10*^+/+^ NOD mice and *BDC2.5*^+^*Il-10*^-/-^ NOD mice.** (**A**) Splenic Treg cell proportion in *BDC2.5*^+^*Il-10*^+/+^ NOD mice and *BDC2.5*^+^*Il-10*^-/-^ NOD mice (*n* = 4-5/group). (**B-C**) Function of splenic Treg cells from *BDC2.5*^+^*Il-10*^+/+^ NOD mice and *BDC2.5*^+^*Il-10*^-/-^ NOD mice in an antigen-specific (mimotope peptide) Treg suppression assay. The experiments were performed twice (A) or three times (B-C), and consistent results were obtained. Data in (A) are shown as mean ± SD and were analyzed using a two-tailed Student's *t*-test. Data in (B-C) were analyzed using a one-way ANOVA test, followed by a two-tailed Student's *t*-test between groups. *P* < 0.05 was considered statistically significant.

**Figure S9.** Immune cells in the intestine of *BDC2.5*^+^ *Il-10*^+/+^ NOD mice and *BDC2.5*^+^*Il-10*^-/-^ NOD mice (*n* = 4-8/group). (**A**) CD11c^+^ cells. (**B**) CD4^+^IFN-γ^+^ cells. (**C**) CD4^+^IL-17^+^ cells. (**D**) TCR-γδ^+^ cells. (**E**) NKP46^+^ cells. (**F**) CD117^+^ cells. Cells were gated from CD45^+^ immune cells. Data are shown as mean ± SD and were analyzed using a two-tailed Student's *t*-test. *P* < 0.05 was considered statistically significant.

**Figure S10. The effects of antibiotic treatment on neutrophils from *BDC2.5*^+^*Il-10*^+/+^ NOD mice. (A)** *BDC2.5*^+^*Il-10*^+/+^ NOD mice showed altered percentages of splenic neutrophils after antibiotic treatment (*n* = 6-9/group)**.** CD11b^+^Ly6G^+^ neutrophils were gated from TCRβ^-^ cells. *P* < 0.05 was considered significant.
